# Influence of a Multicomponent Supervised Exercise Program on Frail and Prefrail Community-Dwelling Older Adults: Protocol for a Randomized Controlled Trial

**DOI:** 10.2196/93130

**Published:** 2026-08-18

**Authors:** Ana Paula Felix Arantes, Fabiana Machado Pires, Caroline Brand, Rodrigo Franco Oliveira, Deise Aparecida de Almeida Pires Oliveira

**Affiliations:** 1 Department of Graduate Studies in Human Movement and Rehabilitation, Universidade Evangelica de Goiás, Av. Universitária Km. 3,5 - Cidade Universitária, Anapolis, Goiás, 75083-515, Brazil, 55 62999758050; 2 Universidade de Rio Verde, Rio Verde, Goiás, Brazil; 3 IRys Group, Physical Education School, Pontificia Universidad Católica de Valparaíso, Valparaiso, Valparaíso, Chile

**Keywords:** aging, exercise, frailty, randomized control trial, protocol

## Abstract

**Background:**

Frailty and prefrailty are highly prevalent conditions among older adults and are associated with increased functional decline, fall risk, hospitalization, and mortality. Multicomponent supervised exercise programs have demonstrated efficacy in improving physical performance and mitigating frailty, particularly when adapted to older adults’ functional capacity. However, evidence regarding Vivifrail-based interventions for frail and prefrail older adults in Brazil remains limited.

**Objective:**

This study aims to evaluate the effects of a 12-week supervised multicomponent exercise program on functional capacity and fall risk among frail and prefrail older adults. Additionally, the study aims to characterize participants according to frailty status, clinical-functional vulnerability, cognitive status, depressive symptoms, physical activity level, muscle mass, fear of falling, and sociodemographic and clinical characteristics at baseline.

**Methods:**

This study protocol describes a prospective, parallel-group, single-blind randomized controlled trial. Older adults aged 60 years and older who regularly attend activities at the CONVIVER Community Center in Rio Verde, Goiás, Brazil, will be screened and randomized in a 1:1 ratio into either an intervention group or a control group. The intervention group will participate in a supervised multicomponent exercise program based on the Vivifrail model for 12 weeks, whereas the control group will participate in health education workshops focused on healthy aging. The primary outcomes will be functional capacity, assessed using the 6-Minute Walk Test, and fall risk and mobility performance, assessed using the Timed Up and Go Test. Baseline assessments will additionally include frailty status (Edmonton Frailty Scale), Clinical-Functional Vulnerability Index-20, cognitive status (Mini-Mental State Examination), depressive symptoms (Geriatric Depression Scale-15), physical activity level (International Physical Activity Questionnaire), calf circumference, fear of falling (Falls Efficacy Scale-International), and sociodemographic and clinical characteristics.

**Results:**

Recruitment and baseline assessments are planned to occur between July and December 2026 at the CONVIVER Community Center. A total of 70 participants are expected to be enrolled and randomized into the intervention group (n=35) or the control group (n=35). At the time of manuscript submission, participant recruitment had not yet started, and no outcome data had been collected or analyzed. Final results are expected to be published in late 2027.

**Conclusions:**

This randomized controlled trial protocol describes a supervised multicomponent exercise intervention tailored to frail and prefrail older adults in a Brazilian community setting. If effective, the intervention may represent a feasible, low-cost, and scalable strategy to improve functional capacity and reduce fall risk in vulnerable older populations while supporting evidence-based healthy aging initiatives.

## Introduction

The global demographic transition toward an aging population represents one of the most significant public health challenges of the 21st century. By 2050, the number of individuals aged 60 years and older is expected to exceed 2.1 billion, with the greatest growth occurring in low- and middle-income countries [1]. This demographic shift is accompanied by an increased prevalence of age-related conditions, including frailty, sarcopenia, functional decline, and falls, which negatively affect independence, quality of life, and health system sustainability [2].

Functional decline and fall risk are particularly relevant public health concerns. Falls are the second leading cause of accidental injury-related deaths worldwide and are associated with substantial physical and psychosocial consequences [3]. Factors such as muscle weakness, postural instability, and sensory deficits contribute significantly to fall risk, highlighting the need for effective preventive strategies [4].

Frailty and prefrailty are key indicators of vulnerability in older adults. Frailty is a multidimensional geriatric syndrome characterized by reduced physiological reserves across multiple systems, increasing susceptibility to stressors such as illness and environmental challenges [5]. Prefrailty represents an intermediate and potentially reversible stage preceding frailty. Among older adults, frailty affects approximately 10% to 15%, while prefrailty may occur in up to 50% [6]. Both conditions are associated with adverse outcomes, including disability, hospitalization, institutionalization, and mortality [7], and are linked to systemic inflammation, immune dysregulation, and sarcopenia [8,9].

Frailty is also strongly associated with an increased risk of falls [3]. In Brazil, falls constitute a major public health issue and are among the leading causes of hospitalization in individuals aged 65 years and older [10]. Given their multifactorial nature, frailty and fall risk require multidimensional preventive approaches. Physical exercise is one of the most effective nonpharmacological strategies for reducing frailty, improving physical function, and promoting healthy aging [11,12]. Regular physical activity reduces chronic disease risk and mortality, whereas sedentary behavior increases the likelihood of dependence, falls, and fractures [13].

Multicomponent exercise programs that combine aerobic, strength, balance, and flexibility training are particularly effective in older populations [13]. These programs improve muscle strength, mobility, postural control, and cardiorespiratory capacity, thereby reducing functional decline and fall risk [14,15]. However, despite growing evidence, studies vary considerably in design and target populations, limiting their generalizability [16,17]. Group-based and supervised interventions appear to enhance adherence and functional outcomes, especially among frail individuals [18-20].

The Vivifrail program, developed within the European Erasmus+ initiative, provides an individualized, function-based exercise prescription tailored to older adults’ performance levels [21]. Evidence suggests that Vivifrail-based interventions can reverse frailty, improve cognition and mood, and prevent functional decline related to hospitalization [22-24]. Nevertheless, its application in Latin America, particularly in Brazilian community settings, remains limited, and randomized controlled trials (RCTs) in this context are scarce [20].

To address these gaps, this study aims to evaluate the effects of a 12-week supervised multicomponent exercise program on functional capacity and fall risk among frail and prefrail older adults. In addition, the study described in this protocol aims to characterize participants according to frailty status, clinical-functional vulnerability, cognitive status, depressive symptoms, physical activity level, muscle mass, fear of falling, and sociodemographic and clinical characteristics at baseline. Its findings aim to support the development of evidence-based, culturally tailored interventions to promote healthy aging in these populations.

## Methods

### Study Design

This study is a prospective, parallel-group, single-blind RCT. The study will be conducted in accordance with the SPIRIT (Standard Protocol Items: Recommendations for Interventional Trials) guidelines. Ethical approval was obtained from the Ethics and Research Committee of the University of Rio Verde (6,658,796; February 20, 2024), and all procedures will follow the ethical principles of the Declaration of Helsinki and its subsequent amendments. Written informed consent will be obtained from all participants prior to enrollment. The clinical trial was prospectively registered with the Brazilian Registry of Clinical Trials under number RBR-9zvtc5b on June 26, 2024.

This protocol was submitted before the initiation of participant recruitment, intervention implementation, data collection, and outcome analysis. At the time of manuscript submission, no study outcomes had been collected or analyzed. The protocol publication aims to ensure methodological transparency, reproducibility, and external peer review before the completion of the clinical trial.

**Figure 1.**
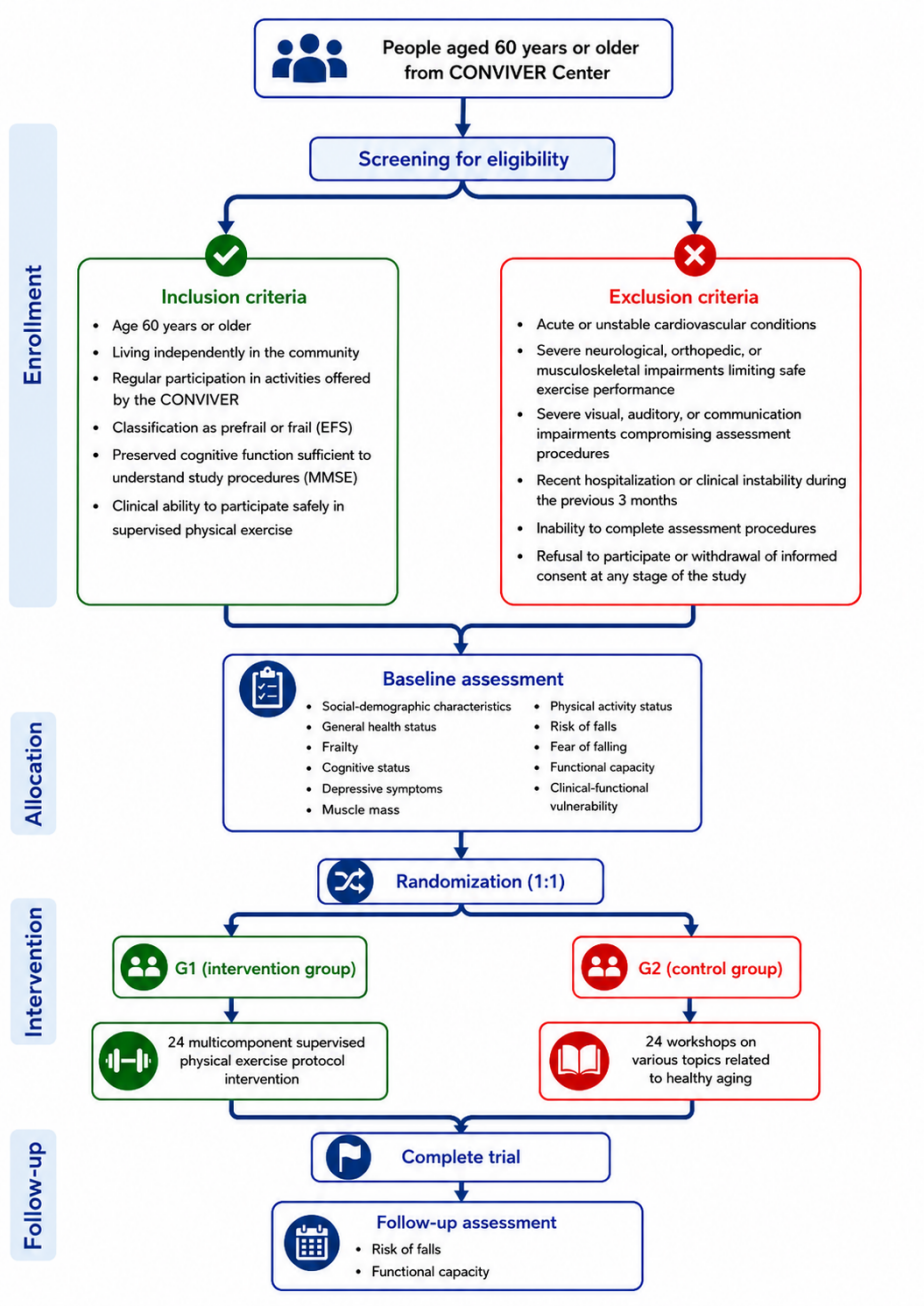
Flow diagram of the study protocol. EFS: Edmonton Frailty Scale; MMSE: Mini-Mental State Examination.

The trial will be conducted at the CONVIVER Community Center for Older Adults, a publicly funded facility located in Rio Verde, Goiás, Brazil, which provides free physical, social, and health-promotion activities for older adults. Participants will be randomly allocated to one of the two groups: (1) a supervised multicomponent exercise group based on the Vivifrail protocol or (2) a control group receiving twice-weekly health education workshops focused on healthy aging topics. The study flow diagram is presented in Figure 1.

### Screening and Recruitment

Screening, recruitment, and baseline assessments are planned to occur between July and December 2026 at the CONVIVER Center facilities. Recruitment will occur exclusively at the center during routine activities regularly attended by older adults already enrolled in the institution. Potential participants will be approached by members of the research team and invited to receive detailed information regarding the objectives, procedures, risks, and benefits of the study.

### Participants

Participants will consist of older adults who regularly attend activities offered by the CONVIVER Community Center for Older Adults in Rio Verde, Goiás, Brazil. The CONVIVER Center is a publicly funded community facility coordinated by the municipal government that provides social, educational, recreational, and health-promotion activities for independently living older adults from the local community.

Individuals who express interest in participating will undergo eligibility screening and baseline assessments after signing the written informed consent form. During the screening phase of the study, frailty status will be assessed to determine the participants’ eligibility.

Inclusion criteria will include (1) age of 60 years or older; (2) living independently in the community; (3) regular participation in activities offered by the CONVIVER Community Center; (4) classification as prefrail or frail according to the Edmonton Frailty Scale (EFS); (5) preserved cognitive function sufficient to understand the study procedures, as assessed using the Mini-Mental State Examination (MMSE); and (6) clinical ability to participate safely in supervised physical exercise activities.

Exclusion criteria will consist of (1) acute or unstable cardiovascular conditions; (2) severe neurological, orthopedic, or musculoskeletal impairments limiting safe exercise performance; (3) severe visual, auditory, or communication impairments compromising assessment procedures; (4) recent hospitalization or clinical instability within the previous 3 months; (5) inability to complete the assessment procedures; and (6) refusal to participate or withdrawal of informed consent at any stage of the study.

Frailty status will be determined using the EFS. Participants scoring between 5 and 6 points will be classified as prefrail, whereas those scoring 7 points or higher will be classified as frail. Individuals scoring between 0 and 4 points, indicating the absence of frailty, will not be eligible for participation in the study [25,26]. Participants attending fewer than 75% of intervention sessions will be considered nonadherent for per-protocol analyses but will remain eligible for intention-to-treat analyses whenever possible.

### Randomization

After the completion of eligibility screening and baseline assessments, participants will be randomly allocated in a 1:1 ratio to either the supervised multicomponent exercise group or the health education control group. Before randomization, participants will be stratified according to frailty status, classified as prefrail or frail based on the EFS, to promote a balanced distribution of frailty levels between groups.

The randomization process will be performed using the online platform Research Randomizer by an independent researcher who will not participate in participant recruitment, assessments, intervention delivery, or data analysis [27]. Allocation concealment will be ensured through sequentially numbered, opaque, sealed envelopes prepared in advance by the independent researcher.

After baseline assessments, the next sequential envelope will be opened by a research assistant who is not involved in outcome assessment procedures. Participants will then be informed of their assigned group and referred to the corresponding intervention procedures.

### Blinding

This study is characterized as a single-blind RCT with blinded outcome assessment. Participants and physiotherapists responsible for the supervised exercise sessions and health education workshops cannot be blinded to group allocation. However, outcome assessors and data analysts will remain blinded throughout the study to minimize assessment and analysis bias. The researcher responsible for the randomization process will not participate in participant recruitment, baseline or follow-up assessments, intervention delivery, or statistical analyses. Participants will be instructed not to disclose their group allocation to the assessors during the evaluation procedures.

### Interventions

#### Study Procedures

Both groups (intervention and control) will undergo baseline and postintervention testing in a randomized order to ensure an unbiased assessment of the outcomes. Participants in both groups will be instructed to continue with their usual care routines and daily activities throughout the duration of the trial.

#### Intervention Group

**Figure 2.**
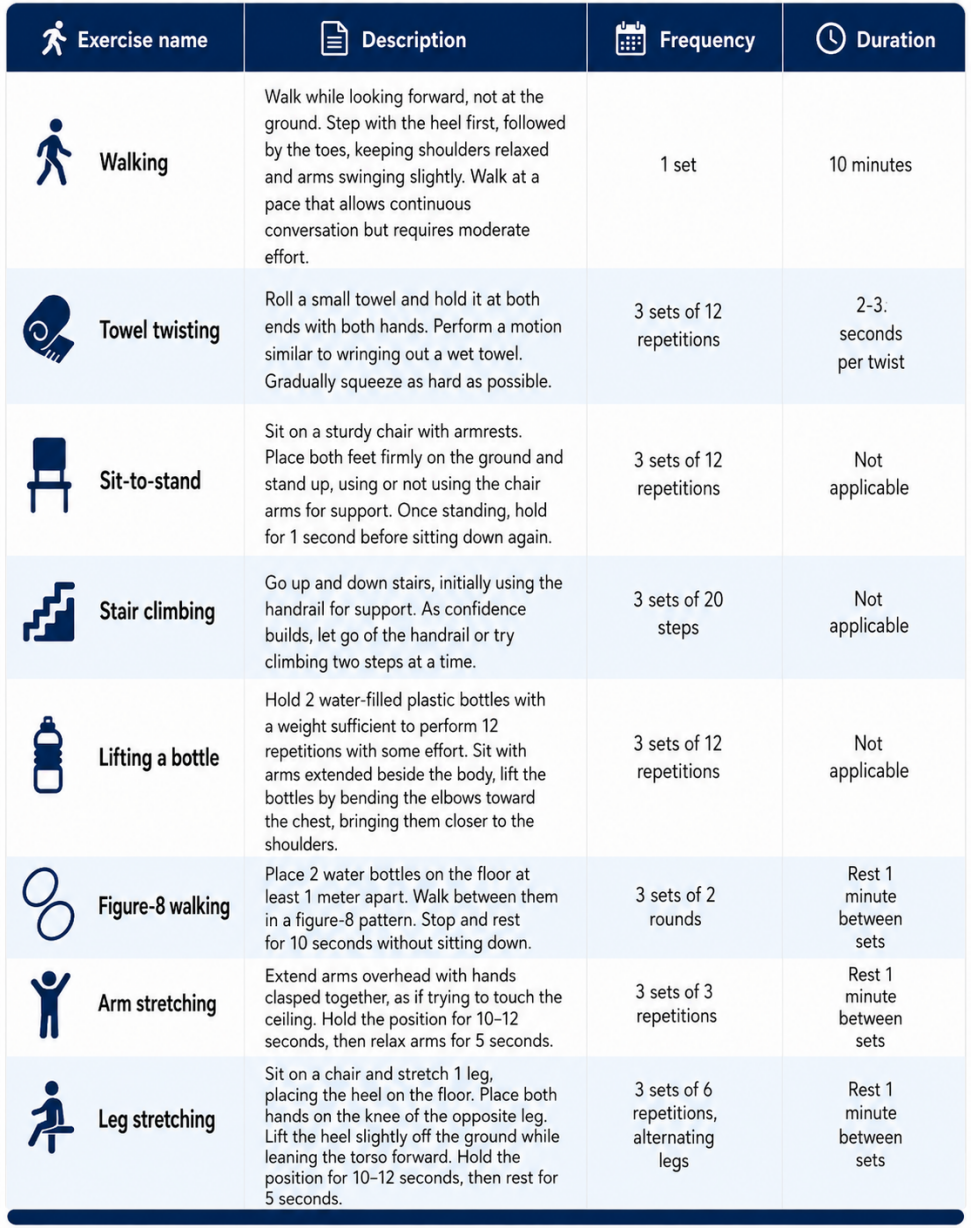
Exercises used in group 1 (intervention) based on the Vivifrail protocol [21].

Participants in the intervention group will engage in a supervised physical exercise program based on the Vivifrail protocol [21]. The program will span 12 weeks, with sessions held twice weekly, totaling 24 sessions, each lasting approximately 45 minutes, and will include exercises targeting strength, balance, mobility, gait, flexibility, and functional endurance. The sessions will be conducted in small groups under the direct supervision of trained physiotherapists, as described in Figure 2.

The exercise program will be supervised by trained physiotherapists and will be conducted on-site at the CONVIVER Community Center. Participants will be advised to wear comfortable clothing and footwear and to maintain proper hydration throughout the intervention. Each session will be documented in a field diary, which will record the session date, participant compliance (whether they completed the full series or if the session was interrupted), any adverse events (AEs), and their descriptions.

The frequency of 2 supervised sessions per week was selected based on the previous clinical trials involving frail and prefrail older adults that demonstrated significant improvements in functional performance, balance, mobility, and frailty-related outcomes using similar exercise frequencies. Chittrakul et al [23] reported positive effects of a multicomponent exercise intervention performed twice weekly on fall prevention and quality of life in prefrail older adults. Likewise, Bray et al [22] observed improvements in physical performance and functional capacity using a supervised multicomponent exercise program delivered 2 times per week in prefrail older women. Furthermore, recommendations derived from the Vivifrail program emphasize that the exercise prescription for frail older adults should prioritize safety, adherence, and individualized progression and that clinically meaningful benefits can be achieved with supervised exercise performed at least twice weekly. Therefore, the selected frequency was considered appropriate to balance effectiveness, safety, and adherence in this vulnerable population.

Exercise intensity and progression will be individualized according to the participants’ baseline functional capacity, frailty level, and clinical tolerance. Perceived exertion during exercise sessions will be monitored using the Borg Rating of Perceived Exertion scale, aiming to maintain exercise intensity between 11 and 13 points (light to somewhat hard intensity) [28].

Physiological responses and clinical symptoms, including excessive fatigue, dizziness, pain, dyspnea, and cardiovascular symptoms, will also be monitored throughout the sessions to ensure participant safety. Progression of the exercise program will occur gradually throughout the intervention period according to individual adaptation and tolerance. Strength exercises will be progressed by increasing the number of repetitions when participants demonstrate adequate performance and safety. Balance and mobility exercises will be progressed through the reduction of external support.

To ensure intervention fidelity and reproducibility, all physiotherapists involved in the intervention will receive standardized training regarding exercise prescription, progression criteria, monitoring procedures, and safety management before the beginning of the trial. Standardized exercise protocols and progression checklists will be used throughout the intervention period. Attendance will be recorded at each session, and adherence rates will be monitored continuously. Any adaptations, interruptions, AEs, or deviations from the intervention protocol will be systematically documented by the research team.

#### Control Group

Older adults allocated to the control group will participate in a series of workshops focused on healthy aging–related topics. These workshops will be conducted on-site at the CONVIVER Community Center and will provide educational content and practical guidance aimed at promoting health, autonomy, self-care, and quality of life among older adults. Topics addressed during the workshops will include healthy lifestyle habits, fall prevention, chronic disease management, nutrition, medication use, mental health, sleep quality, and strategies to maintain independence during aging.

**Figure 3.**
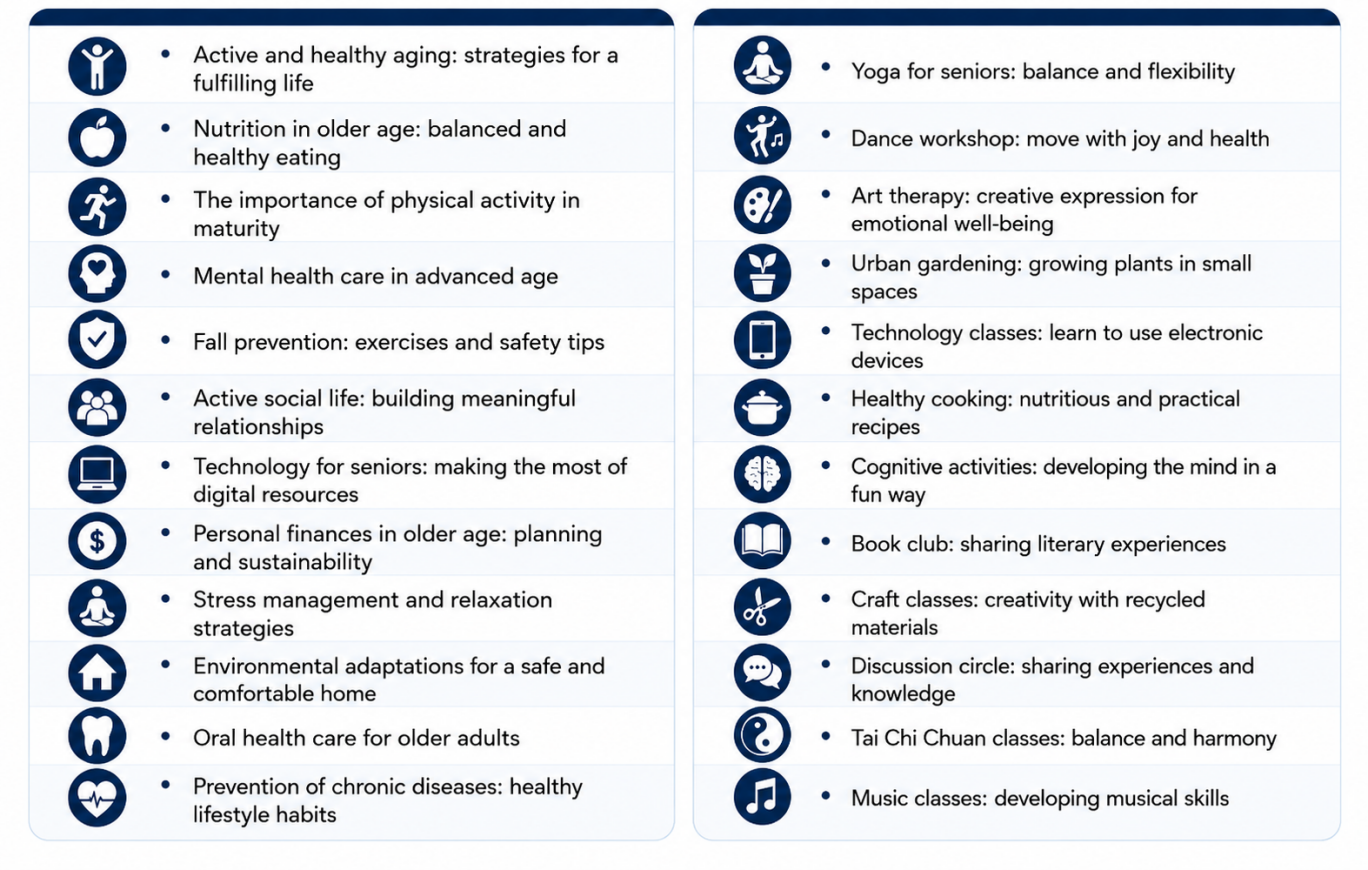
Topics of lectures and workshops conducted in the control group.

The workshops will occur twice weekly in parallel with the intervention group activities, with each session lasting approximately 45 minutes (Figure 3). This comparator was selected because the CONVIVER center routinely offers health education and social support activities as part of standard community care for older adults. Therefore, participants allocated to the control group will continue receiving usual community-based care and social interaction throughout the study period.

The use of an educational control group was chosen to allow for a comparison between a structured supervised exercise intervention and a nonexercised intervention focused on health promotion, while minimizing complete inactivity and maintaining participant engagement during the trial. This strategy also seeks to reduce ethical concerns related to the withdrawal of social and educational support in a vulnerable older population.

In addition, after the completion of postintervention assessments and study procedures, participants allocated to the control group will be invited to participate in the supervised multicomponent exercise program offered by the research team. This delayed-access strategy was adopted to ensure ethical balance and to provide all participants with the opportunity to benefit from the exercise intervention after the conclusion of the trial.

To monitor potential contamination related to external physical activity during the intervention period, participants from both groups will be instructed not to initiate new structured exercise programs outside the study protocol. Physical activity behavior will be monitored through self-reports.

### Outcome Measurements

#### Assessment Procedures

**Figure 4.**
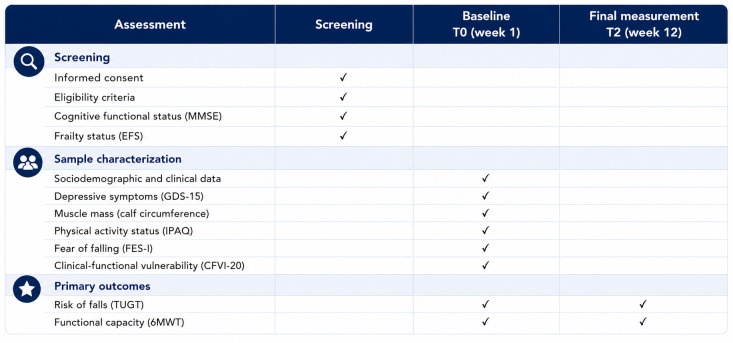
Schedule of assessments for screening, sample characterization, and primary outcomes. CFVI-20: Clinical-Functional Vulnerability Index-20; EFS: Edmonton Frailty Scale; FES-I: Falls Efficacy Scale-International; GDS-15: Geriatric Depression Scale-15; IPAQ: International Physical Activity Questionnaire; MMSE: Mini-Mental State Examination; 6MWT: 6-Minute Walking Test; TUGT: Timed Up and Go Test.

During the screening phase, participants will sign the informed consent form and undergo an eligibility assessment, cognitive screening using the MMSE, and frailty classification using the EFS (Figure 4).

At baseline, sociodemographic and clinical data will be collected, along with assessments of depressive symptoms (Geriatric Depression Scale-15 [GDS-15]), muscle mass (calf circumference), physical activity level (International Physical Activity Questionnaire [IPAQ]), fear of falling (Falls Efficacy Scale-International [FES-I]), and clinical-functional vulnerability (Clinical-Functional Vulnerability Index-20 [CFVI-20]) to characterize the older adults. The primary outcomes, namely functional mobility assessed by the Timed Up and Go Test (TUGT) and functional capacity assessed by the 6-Minute Walk Test (6MWT), will be evaluated at baseline and reassessed after the 12-week intervention period (T2).

#### Screening Measurements

##### Cognitive Functional Status

The MMSE will be used to assess cognitive functional status. It is an instrument designed for the initial screening of mental status to evaluate the presence of cognitive deficits. It includes 2 categories of responses: verbal and nonverbal. The MMSE consists of 30 categorical questions. The original scoring of the test classifies cognitive impairment as follows: 30 to 26 (preserved cognitive function); 26 to 24 (suggestive alterations of cognitive impairment); and 23 or below (cognitive impairment). For this study, a cutoff score of 19 will be applied [29-31].

##### Frailty Status

Frailty status will be assessed using the EFS, a multidimensional instrument validated for use in older adults. The scale evaluates domains including cognition, general health status, functional independence, social support, medication use, nutrition, mood, continence, and functional performance [25]. Scores range from 0 to 17 points, with higher scores indicating greater frailty. In this study, participants scoring 5 to 6 points will be classified as prefrail, whereas those scoring ≥7 points will be classified as frail. Individuals scoring 0 to 4 points will be considered nonfrail and will not be included in the trial [26].

### Other Measurements

#### Sociodemographic and Clinical Data

Sociodemographic and economic data, along with general health and lifestyle habits, were collected using a structured form developed by the researchers. This section of the assessment includes various items aimed at capturing key aspects of the participants’ personal and health-related characteristics. The form includes the following data points: name, date of birth, race, marital status, number of children, education level, source of income, monthly income in terms of minimum wage, self-perceived health status, self-reported chronic diseases, presence of depressive symptoms (assessed using the GDS-15), and muscle mass (measured using calf circumference).

#### Depressive Symptoms

The emotional state of participants will be evaluated using the GDS-15 [32,33]. The GDS-15 consists of 15 questions with “Yes” or “No” responses, scored 0 or 1 depending on the question. The total score ranges from 0 to 15 points. In this study, symptoms of depression will be considered present in individuals scoring 6 or more points.

#### Muscle Mass

This will be evaluated through calf circumference measurement. The technique involves asking the participant to stand upright with feet 20 cm apart. Using a nonelastic tape measure, the maximum circumference of the calf is measured perpendicular to its longitudinal axis. Values below 34 cm in men and 33 cm in women indicate reduced muscle mass [34,35]

#### Physical Activity Status

The IPAQ will be applied at baseline to characterize participants’ habitual physical activity levels, and postintervention measurements will be used exclusively for contamination monitoring and not as efficacy outcomes. It consists of 8 open-ended questions about the time spent on moderate- and vigorous-intensity physical activities and sedentary activities (sitting) in a typical week [36]. Based on the IPAQ scoring protocol, participants will be classified into 4 physical activity levels according to the frequency, duration, and intensity of weekly physical activity: inactive, insufficiently active, active, or very active.

Participants classified as inactive will report no regular physical activity lasting at least 10 continuous minutes during the week. Individuals classified as insufficiently active will report some physical activity but will not achieve the minimum recommended levels. Participants classified as active will meet the recommendation of at least 150 minutes of moderate-intensity physical activity per week, 75 minutes of vigorous-intensity activity per week, or an equivalent combination of both. Those classified as very active will exceed the minimum recommended physical activity levels according to the IPAQ scoring criteria [37].

#### Fear of Falling

Fear of falling will be evaluated using the FES-I. The scale comprises 16 questions addressing concerns about falling during daily tasks such as cleaning, cooking, reaching for objects on the floor or overhead, and climbing stairs or ramps. Scores range from 16 to 64, where 16 indicates no concern about falling, and scores above 17 suggest some degree of concern [38-40].

#### Clinical-Functional Vulnerability

Functional decline vulnerability will be assessed using the CFVI-20 [41]. This instrument comprises 20 questions across 8 sections: age, self-perceived health, functional disability, cognition, mood, mobility, communication, and comorbidities. Scores range up to 40, with higher values indicating greater vulnerability. Classifications are as follows: 0‐6 (robust), 7‐14 (potentially frail), and 15+ (frail).

### Primary Outcomes

#### Risk of Falls

Risk of falls will be assessed using the TUGT. The test requires a stopwatch, a chair, and a measuring tape. Participants sit on a chair, and the procedure is explained. They are asked to stand, walk to a line 3 meters away at a self-selected but safe pace, turn around, walk back, and sit down again. Lower times indicate better performance and ≤20 seconds indicates risk of falls [42-44].

#### Functional Capacity

Functional capacity will be measured by the 6MWT following the American Thoracic Society (ATS) protocol [45]. After completion, the same measurements will be repeated, and the total distance walked will be recorded. This procedure will occur pre intervention and post intervention, and results will be compared to a predicted value to assess changes in functional capacity: 6MWD (6-minute walk distance) = 890.46 – (6.11 × age) + (0.0345 × age²) + (48.87 × gender) – (4.87 × BMI), where the 6MWD equation estimates walking distance in meters based on age (in years), gender (1=male, 0=female), and BMI (kg/m²) [46]. It accounts for age-related decline, sex differences, and body composition effects on functional capacity [47].

### Sample Size

Sample size estimation was performed using G*Power software (version 3.1.9.7; Heinrich Heine University) [48,49]. The calculation was based on the primary outcomes of functional capacity and fall risk, assessed by the 6MWT and the TUGT, respectively. An effect size of 0.56 was adopted based on the findings of Espejo-Antúnez et al [50], who evaluated the effects of a 12-week exercise intervention on functional capacity and fall-related outcomes in older adults. Assuming a 2-group parallel RCT design, a statistical power of 95%, and a significance level of 5% (*α*=.05), the minimum required sample size was estimated at 35 participants per group. Therefore, the study was designed to include 70 participants, with 35 allocated to each group. Because recruitment will be conducted among community-dwelling older adults regularly attending activities at the CONVIVER Community Center, and eligibility will depend on screening procedures, the final sample will be obtained through convenience sampling. Recruitment feasibility was considered during the planning phase to ensure attainment of the target sample size.

### Statistical Analysis

For the statistical analysis, data normality will be assessed using the Shapiro-Wilk test and Q-Q plots. Descriptive statistics will be performed to characterize the sample. Categorical variables will be presented as absolute and relative frequencies, whereas continuous variables will be expressed as means and SDs or medians and IQRs, in addition to graphical representations. Changes in mean values over time will be represented by delta values (Δ).

The primary analytical model for the 2 primary outcomes (6MWT and TUGT) will be an analysis of covariance, comparing postintervention values between groups while adjusting for the corresponding baseline values. Preintervention and postintervention comparisons will be conducted using the 2-tailed paired Student *t* test for normally distributed variables or the Wilcoxon signed-rank test for nonnormally distributed variables. Between-group differences will be analyzed using the independent Student *t* test for normally distributed variables and the Mann-Whitney *U* test for nonparametric variables.

Effect sizes will be calculated using eta squared (η²) and Cohen *d* and interpreted as small (0.2), medium (0.5), or large (0.8). All losses to follow-up and their respective treatment durations will be recorded for intention-to-treat analysis. All participants will be reassessed after the 12-week intervention period whenever possible. Missing data will initially be explored to determine the pattern and mechanism of missingness. When appropriate, missing outcome data will be handled using multiple imputation methods based on chained equations. Sensitivity analyses will be conducted to compare the results obtained from complete-case analyses and imputed datasets in order to evaluate the robustness and consistency of the findings. In addition, per-protocol analyses will be performed as secondary sensitivity analyses to assess the influence of adherence to the intervention on study outcomes. A significance level of *P*<.05 will be adopted for all statistical tests. Statistical analyses will be performed using the SPSS, version 25.0 (IBM Corp) [51], and graphs will be generated using R software (R Foundation for Statistical Computing) [52].

### Safety Assessment and Withdrawal

Participant safety will be monitored throughout the intervention period by the research team and supervising physiotherapists. Any AEs potentially associated with the exercise sessions or assessment procedures, including dizziness, pain, excessive fatigue, musculoskeletal discomfort, falls, cardiovascular symptoms, or any other clinical complaints, will be immediately documented and evaluated.

If a participant develops any AE or clinical instability during the intervention period, exercise participation will be temporarily interrupted, and the participant will be closely monitored by the research team. When necessary, the participant will be referred for medical evaluation before resuming study activities. In cases where symptoms persist, recur after reintroduction of the intervention, or compromise participant safety, the participant may be withdrawn from the study after clinical assessment by the investigators. All AEs and safety-related occurrences will be systematically recorded in standardized monitoring forms throughout the trial period.

Participants will be free to discontinue their participation at any moment and for any reason, including the option to request the removal of their data from the study. Individuals will be considered withdrawn if they formally request to leave the trial or are lost to follow-up. Participants allocated to the intervention group will be classified as withdrawn if they discontinue participation in the supervised exercise program. Participants allocated to the control group will be classified as withdrawn if they discontinue participation in the educational workshops before the completion of the study.

### Data Monitoring

The data that support the findings of this study will not be publicly available due to information that could compromise the privacy of research participants. Upon reasonable request, data sharing will be considered by the corresponding author. The principal investigator will enter the collected information into a computerized database. Participants’ identities will be anonymized and replaced with coded identifiers. All study-related data will be safely stored on encrypted computer systems at the research center, with access restricted exclusively to members of the research team, archived, and retained for a period of up to 5 years.

### Ethical Considerations

This study was approved by the Research Ethics Committee of the University of Rio Verde, Brazil (process number 77067323.1.0000.5077 and approval number 6.658.796). Its results will be disseminated through multiple channels, including presentations at international conferences and publication in peer-reviewed journals. The findings will be reported in accordance with the SPIRIT (Standard Protocol Items: Recommendations for Interventional Trials) guidelines, ensuring transparency and adherence to best practices in clinical research. Written informed consent was secured from all participants prior to the baseline assessments. After the completion of follow-up data collection, and in accordance with participants’ stated preferences and needs, individuals allocated to the control group will be offered access to the intervention components and will receive the material support not previously provided. The conduct of this study will strictly follow the ethical principles of beneficence, respect for human dignity, justice, and integrity in the reporting of truth and facts.

## Results

The current status and projected timeline of the trial are presented herein. Recruitment and baseline assessments are scheduled to be conducted between July and December 2026 at the CONVIVER Community Center in Rio Verde, Goiás, Brazil, among older adults regularly attending activities at the center. A total of 70 eligible participants are expected to be enrolled and randomized into the intervention group (n=35) or the control group (n=35). The intervention and postintervention assessments are expected to be completed in early 2027. At the time of manuscript submission, participant recruitment has not yet started. Statistical analyses and dissemination of the results are expected to occur in late 2027. This protocol publication aims to ensure methodological transparency and facilitate peer review of the study design and procedures before the publication of the primary outcomes.

## Discussion

### Anticipated Findings

This study protocol describes an RCT designed to examine the effects of a supervised multicomponent exercise program, adapted from the Vivifrail model, on frailty status, functional capacity, clinical-functional vulnerability, and fall risk among frail and prefrail older adults in Brazil. The proposed intervention integrates aerobic, strength, balance, and flexibility exercises within a structured, individualized, and supervised framework, aiming to enhance functional performance and promote active aging.

It is expected that the 12-week supervised multicomponent exercise program will lead to significant improvements in frailty status, functional capacity, clinical-functional vulnerability, and fall risk among frail and prefrail older adults. Participants in the intervention group are anticipated to show reductions in frailty scores on the EFS, reflecting a shift toward more robust functional profiles. Improvements in physical performance, as measured by the 6MWT and TUGT, are expected to indicate enhanced functional capacity and reduced fall risk. Additionally, positive changes in sarcopenia markers (eg, increased calf circumference), lower fear of falling, and improved mood and cognitive function are also anticipated.

Multicomponent exercise programs, which include strength, balance, aerobic, and flexibility exercises, have demonstrated efficacy in addressing key aspects of frailty [22]. Resistance training improves muscle strength and mitigates sarcopenia, while balance and flexibility exercises enhance postural control and reduce the risk of falls [23]. Aerobic exercise contributes to cardiovascular health and functional endurance, promoting independence in activities of daily living [53]. Previous studies have highlighted the benefits of such programs in reducing frailty levels, improving mobility, and increasing the quality of life in older adults [54-56].

The protocol outlined in this study includes a comprehensive range of exercises designed to address frailty and its associated risks holistically. Activities such as multidirectional walking, stepping over obstacles, sit-to-stand transitions, and balance tasks on stable and unstable surfaces aim to improve muscle activation, postural control, and functional capacity [57]. Furthermore, the inclusion of cognitive-motor tasks, such as walking while performing dual tasks, seeks to enhance executive function and reduce fall risk, which are critical for this population [58].

Despite all these, this study protocol may present several potential limitations. First, the single-center design could limit the generalizability of the findings to other populations or settings. The relatively small sample size, although adequately powered for the primary outcomes, might reduce the ability to detect smaller effect sizes or subgroup differences. Additionally, the expected predominance of female participants, which reflects the demographic profile of the community center, could restrict the applicability of the results to older male adults. Finally, the absence of long-term follow-up will limit conclusions regarding the sustainability of the intervention’s effects over time. These potential limitations should be considered when interpreting the future findings and in the design of subsequent studies.

If proven effective, this supervised multicomponent intervention may serve as a practical and accessible public health approach to promote functional resilience and reduce fall-related morbidity in vulnerable older populations. Ultimately, the findings from this study may inform evidence-based recommendations and guide future exercise programs and health policies aimed at improving the quality of life and well-being of frail and prefrail older adults living in the community.

### Strengths and Limitations

This study presents some limitations that may impact the generalizability of its results. The selection of participants through convenience sampling, restricted to older adults attending the CONVIVER Community Center, may not represent the broader older population of Rio Verde. Additionally, the exclusion criteria, such as limiting participation to individuals with some degree of frailty, restrict the applicability of the findings to a specific group. Voluntary participation may introduce bias, favoring individuals who are more motivated, which could influence the intervention outcomes. Another factor to consider is the intervention frequency, conducted only twice a week, which may reduce the program’s effects compared to protocols with a higher training volume. Furthermore, the study does not strictly control external factors, such as other physical activities performed by participants, which could impact the results. Although randomization is stratified by frailty status to reduce imbalance between groups, residual imbalance in other baseline characteristics, such as age, sex, comorbidities, and functional capacity, may still occur due to the relatively small sample size and single-center design.

Another potential limitation is the possibility of contamination between groups. Because participants will be recruited from the same community center and may interact during routine activities outside the study sessions, information and experiences related to the interventions may be shared between participants allocated to different groups. Although educational workshops and exercise sessions will be conducted separately and participants will be instructed not to discuss the intervention content with other participants, some degree of contamination cannot be completely excluded. This may reduce the observable differences between groups and potentially underestimate the true effect of the intervention.

On the other hand, the study has strengths that reinforce its validity and relevance. The intervention follows the Vivifrail protocol, a structured and scientifically supported program, ensuring methodological consistency. Supervision by physiotherapists contributes to the safety and proper execution of the exercises, reducing risks and improving adherence. The randomized controlled design enhances internal validity, allowing for more robust inferences about the intervention’s effects. The use of field diaries to monitor adherence, interruptions, and AEs improves participant follow-up. Additionally, the clear definition of inclusion and exclusion criteria, with validated instruments such as the MMSE and the EFS, ensures a more homogeneous sample. The 12-week duration, with 24 training sessions, is adequate to assess significant functional changes in frail older adults. Lastly, the adoption of a single-blind design helps minimize biases in outcome assessments, strengthening the reliability of the results.

## Abbreviations

6MWT: 6-Minute Walk Test
AE: adverse event
ATS: American Thoracic Society
CFVI-20: Clinical-Functional Vulnerability Index-20
EFS: Edmonton Frailty Scale
FES-I: Falls Efficacy Scale-International
GDS-15: Geriatric Depression Scale-15
IPAQ: International Physical Activity Questionnaire
MMSE: Mini-Mental State Examination
RCT: randomized controlled trial
SPIRIT: Standard Protocol Items: Recommendations for Interventional Trials
TUGT: Timed Up and Go Test
